# 3D Numerical Simulation of Reactive Extrusion Processes for Preparing PP/TiO_2_ Nanocomposites in a Corotating Twin Screw Extruder

**DOI:** 10.3390/ma12040671

**Published:** 2019-02-23

**Authors:** Dapeng Sun, Xiangzhe Zhu, Mingguang Gao

**Affiliations:** 1 School of Mechanical Engineering, Liaoning Shihua University, Fushun 113001, China; xzzhu@126.com; dpsun2019@163.com (D.S.); mggao1987@163.com (M.G.); 2 College of Pipeline and Civil Engineering, China University of Petroleum (East China), Qingdao 266580, China

**Keywords:** twin screw extruder, simulation, residence time distribution, PP/TiO_2_ nanocomposites, conversion

## Abstract

To better understand the relationship between flow, mixing and reactions in the process of preparing PP/TiO_2_, a 3D numerical simulation in a co-rotating twin screw extruder (TSE) was firstly employed using commercial CFD code, ANSYS Polyflow. The effects of rotating speed of screws, stagger angle of knead blocks, inlet flow rate and initial temperature of barrel on the mixing and reaction process in the TSE were investigated. The results reveal that the studied operational and geometric parameters, which determine mixing efficiency, residence time distribution, and temperature of the flows in the TSE, affect the local species concentration, reaction time and reaction rate, and hence have great influences on the conversion rate. The results show that increasing the rotating speed and inlet flow rate can decrease the time for sufficient mixing, which is not conducive to intensive reaction, and increasing the stagger angle has the opposite effect. Moreover, the conversion rate greatly affected by the initial temperature of barrel.

## 1. Introduction

The application of Polypropylene (PP) covers many industrial fields, including film, thermoforming and automotive fields, due to its easy processability, relatively high mechanical properties and great recyclability [1]. Nevertheless, there are disadvantages of net PP, such as low polarity, poor ultraviolet, low thermal stability and non-bacterial resistance [2]. One way to improve the PP properties mentioned above is to disperse titanium dioxide (TiO_2_) into PP matrix [3]. In situ sol–gel reactions in melt are the most suitable method for producing dispersed TiO_2_ nanoparticles in PP matrix without solvent, and it can be applied to industrial processes in a twin screw extruder. Polymer matrix-based nanocomposites have attracted a lot of attention in the recent literature [4,5]. Meanwhile, twin screw extruders (TSEs) have been broadly applied in the processing of polymer nanocomposites due to their excellent mixing performance and modular design characteristic. With the great improvement of computing power, the simulation methods have been intensively used to better understand the factors influencing the preparation of nanocomposites by reactive extrusion.

The twin screw extruder (TSE) is a key equipment in polymer processing, which is mainly used as a conveyor, mixer or reactor for particles, granules and viscous fluids [6]. As polymer mixing equipment, the mechanism of melt flow and mixing is very complex. With the development of the computational method, more and more researchers are studying the isothermal or non-isothermal flow mechanisms in TSEs by means of numerical simulation [7,8,9,10]. Recently, Zhang et al. [11] analyzed the mixing of generalized Newtonian fluid in kneading zones of a TSE using finite element method (FEM) and experiment. Distributive mixing and overall efficiency of different types of kneading blocks were studied by calculating the mixing parameters, such as area stretch ratio, instantaneous efficiency, and time-average efficiency. Robinson et al. [12] carried out numerical simulations of three-dimensional flow in a co-rotating TSE to reveal the influences of geometry and fill level on the transport and mixing behavior. Reitz et al. [13] studied the residence time distribution of hot melt extrusion processes experimentally and mathematically. Kruijt et al. [14,15,16] proposed a mapping method of polymer melt in a co-rotating TSE in terms of the concentration distribution, residence time distribution and separation strength to characterize the distribution mixing.

Generally, TSE is taken as a continuous reactor in which the reactions and extrusion process occur simultaneously [17,18]. Consequently, reactive extrusion is a complex process that involves many aspects, because of the high number of operating variables and their interactions during the process [19]. For example, with the proceeding of mixing and reaction process, the properties of the molten polymers will change along the axial direction in TSEs. The residence time distribution, pressure, segregation scale and mixing efficiency are important indexes to measure the mixing performances, which also have great influence on the reaction, and that will in turn modify the material properties. Moreover, the reactive extrusion processes are often accompanied by heat transfer, viscous dissipation and reaction heat. 

To investigate the effects of operating parameters and screw geometry on the mixing performance during reactive extrusion process in TSEs, lots of experimental and numerical simulation studies have been employed in recent years [20,21,22]. Typically, Rozen [23] experimentally and numerically studied the product distribution of two parallel chemical reactions to characterize mixing in a corotating TES. The correlation between the rate of viscous deformation of liquid elements and screw rotational speed was investigated. Zhu et al. [24] analyzed the polymerization of e-caprolactone in a co-rotating TSE with finite element method. The effects of screw rotational speed, geometry of screw element, and initial conversion at the channel inlet on polymerization was discussed. Zhang et al. [25] investigated the effects of screw configuration, rotating speed and silica on the mechanical and foaming properties of PP/wood-fiber composites. Kim et al. [26] studied the process of synthesizing polyamide polyester-based diblock, triblock, and random block copolymers in a modular, corotating TSE via reactive extrusion. Rigoussen et al. [27] investigated the effect and role of cardanol in the compatibilization of PLA/ABS immiscible blends by reactive extrusion. Berzin et al. [28,29] investigated the effects of different oxidant concentrations and basic conditions on the molecular weight distribution and rheological properties of PP, then compared the theoretical model with the experimental data.

However, the above studies were focused on the reactive extrusion processes, such as monomer polymerization, polymer chemical modification, rheological modification, etc.; few studies have been conducted on the sol-gel reaction occurring in the polymer matrix from dynamic simulation viewpoint. As for research on PP/TiO_2_ nanocomposites, most of it has focused on the properties of experimental products using several techniques, such as XRD, TEM micrographs, Raman spectroscopy, X-ray diffraction and mechanical analysis [5,30,31]. However, studies on the mixing and reaction process of PP/TiO_2_ nanocomposites by means of numerical simulation based on Computational Fluid Dynamics (CFD) are few and are limited to 1D or 2D models [32,33]. In the present paper, the reaction process of preparing PP/TiO_2_ nanocomposites in a corotating TSE with two combined screws was investigated via 3D numerical simulation based on FEM. The reactive patterns of nanocomposites in the corotating TSE were investigated firstly. Moreover, the effects of screw rotational speed, stagger angle of kneading blocks, inlet flow rate and initial temperature of barrel on the mixing and reaction process were discussed. The aim of this study is to better understand the effects of geometric and operating parameters on the reactive extrusion process and investigate the relationship between the mixing characteristics and the reactive process in a corotating TSE.

## 2. Materials and Methods 

### 2.1. Geometrical and FE Models

**Figure 1:**
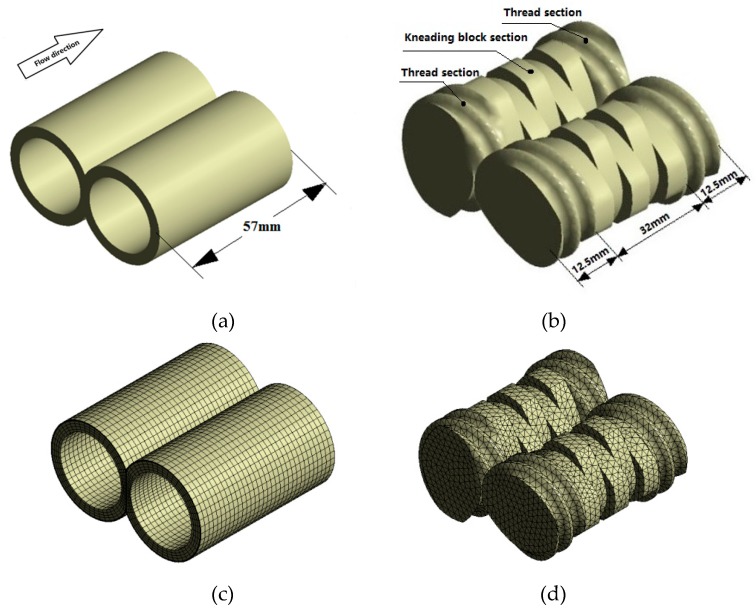
Geometrical and FE models: (**a**) Geometry of flow channel; (**b**) geometry of combined screws; (**c**) FE model of flow channel; (**d**) FE model of two screws.

The corotating TSE investigated in this study consisted of a flow channel and two combined screw elements, as shown in Figure 1a,b, which consisted of thread sections and kneading block sections. Meanwhile, the stagger angle of the kneading block section is 90°, as shown in Figure 1b. The lengths of kneading blocks and thread sections were 32 mm and 25 mm, respectively. Gambit software was used to establish the geometric models of channel and screws, respectively. 

The mesh superposition technique is used to put the meshes of flow channel and screws together without remeshing for the periodical geometric changes. Figure 1c,d illustrates the three-dimensional finite element (FE) models of the flow channel and combined screws of the TSE. The quadrilateral cells are used to mesh the flow channel. To achieve accurate simulation results in the small clearances and near the walls, four boundary layer grids are employed in the FE model of the flow channel. The FE model of the flow channel consists of 34,300 cells and 38,232 nodes. For the FE model of the screws, the tetrahedral cells are used for partition, different screw structures with different stagger angles (see Figure 12) adopt the same meshing method and mesh density. The geometric dimensions of the TSE are listed in Table 1.

### 2.2. Mathematical Models

**Figure 2:**
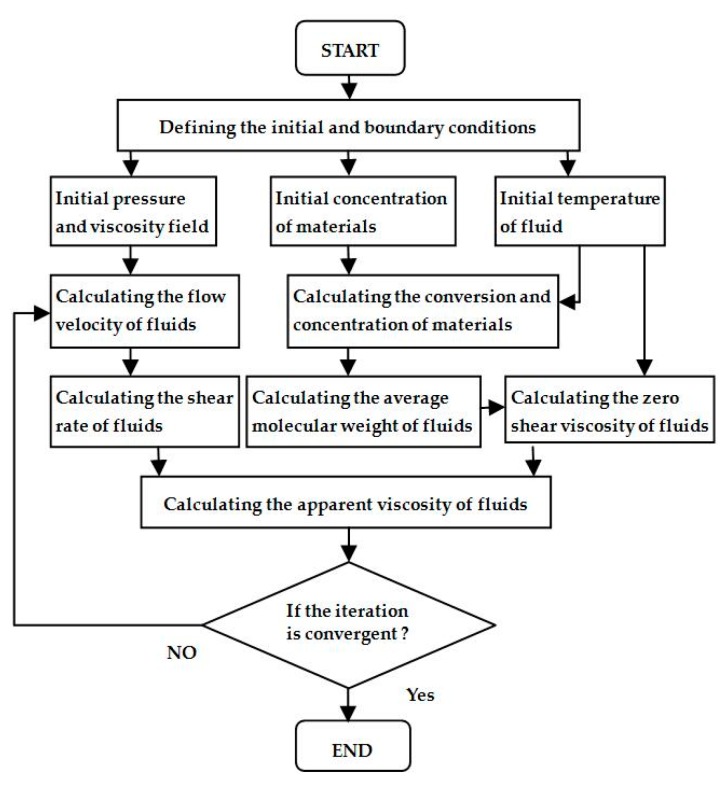
Flow chart of numerical simulation.

Due to the specific conditions of the extrusion process and the characteristics of polymer, the following assumptions are made in order to ensure the accuracy of calculation of the three-dimensional flow field: (1) The channel is fully filled with polymer melt at all times. (2) The fluid is incompressible. (3) The flow is laminar flow. (4) There is no slip between the fluid and the wall. (5) The force of inertia and the force of gravity are ignored. Based on above assumptions, the flow chart of the numerical simulation is shown in Figure 2.

The governing equations used in this paper are as follows:

The continuity and momentum equations are respectively expressed as follows [34]:

(1) ∇⋅V=0

(2) $$\rho \left(\frac{\partial V}{\partial t}+V\cdot \nabla V\right)=-\nabla p+\eta \nabla^{2}V+\rho g$$

where v denotes the velocity vector; *p* denotes the pressure; *ρ* denotes the density, *t* and *η* denotes time, and shear viscosity, respectively.

The energy conservation obeys the following equation [34]:

(3) $$\rho C_{P}\frac{\partial T}{\partial t}+\rho C_{P}V\cdot \nabla T=\tau :\nabla \cdot V+r-\nabla \cdot \left(k\nabla T\right)$$

where *T* denotes the absolute temperature, $C_{P}$ denotes heat capacity, r denotes the heat source, k denotes the kinetic constant, k∇T denotes the heat flux,τ is the stress tensor, the form of τ=2 denotes the viscous heating.

The stress tensor in Equation (3) is written as follows:

(4) $$\tau =2\eta (\dot{\gamma },T)D$$

In which D denotes the deformation tensor rate, and $D=\frac{1}{2}\left[\Delta V+(\Delta V{)}^{T}\right]$; η denotes the shear viscosity; $\dot{\gamma }$ denotes the effective shear rate and can be expressed as:

(5) $$\dot{\gamma }=\sqrt{2\left(D:D\right)}$$

The convection-diffusion equation can be defined as:

(6) $$\frac{\partial }{\partial t}(\rho \omega_{i})+\nabla \cdot (\rho \omega_{i}v)+\nabla \cdot j_{i}=R_{i}+S_{i}$$

where $\omega_{i}$ represents the mass fraction of the component i, $R_{i}$ is the source term of the reaction, $S_{i}$ represents a user-defined source item, $j_{i}$ represents mass diffusion flux.

In this work, it is assumed that the nanocomposites have the viscosity of the PP matrix. This is predicted by the Carreau-Yasuda law:

(7) $$\eta =H(T)\cdot \eta_{0}{\left[1+{\left(\lambda \dot{\gamma }\right)}^{a}\right]}^{\frac{n-1}{a}}$$

where λ is the characteristic time, $\dot{\gamma }$ is the shear rate, a represents the Yasuda parameter, n is the power-law index. According to the Arrhenius law, H(T) can be defined as:

(8) $$H(T)=\exp(\frac{E}{R}(\frac{1}{T}-\frac{1}{T_{r}}))$$

In which *E* is an activation energy for flow, *R* is the gas constant, and *T_r_* is the reference temperature.

### 2.3. Reaction Kinetics

The formation of TiO_2_ in this paper was based on the sol-gel method, and tetrabutyl titanate (Ti(OC_4_H_9_)_4_) was used as the alkyd precursor with high chemical activity. It is assumed that after the precursor material with a mass equivalent to a certain percentage of PP melt is uniformly mixed with PP melt, the precursor material tetrabutyl titanate will undergo hydrolysis and condensation reaction in the PP matrix. The principle is as follows [32]:

(9) $$\mathrm{Ti}{\left(\mathrm{OR}\right)}_{4}+H_{2}O⇌\mathrm{Ti}{\left(\mathrm{OR}\right)}_{3}\mathrm{OH}+\mathrm{ROH}$$

The condensation reactions lead to the formation of oxo bridges, Ti–O–Ti represented by the following reactions:

Alcoxolation or alcohol elimination:

(10) $$\mathrm{Ti}{\left(\mathrm{OR}\right)}_{3}\mathrm{OH}+\mathrm{Ti}{\left(\mathrm{OR}\right)}_{4}⇌{\left(\mathrm{OR}\right)}_{3}-\mathrm{Ti}-O-\mathrm{Ti}-{\left(\mathrm{OR}\right)}_{3}+\mathrm{ROH}$$

Oxalation or water elimination:

(11) $$\mathrm{Ti}{\left(\mathrm{OR}\right)}_{3}\mathrm{OH}+\mathrm{Ti}{\left(\mathrm{OR}\right)}_{3}\mathrm{OH}⇌{\left(\mathrm{OR}\right)}_{3}\mathrm{Ti}-O-\mathrm{Ti}-{\left(\mathrm{OR}\right)}_{3}{+H}_{2}O$$

The overall generalized reaction can be written as:

Ti(OR)_4_ + 2H_2_O ⇌ TiO_2_ + 4ROH (12)

When a quantity of inorganic precursor, Ti(OR)_4_, is introduced into the molten PP matrix, the dilution effect on the PP matrix must be considered in the modeling. Therefore, based on a mixing law, the zero-shear viscosity $\eta_{0}$ of mixing fluid can be defined as:

(13) $$\eta_{0}=\eta_{0\_{\mathrm{Ti}(\mathrm{OR})}_{4}}\left[{\mathrm{Ti}(\mathrm{OR})}_{4}\right]+\eta_{0\_\mathrm{PP}}(1-\left[{\mathrm{Ti}(\mathrm{OR})}_{4}\right])$$

where $\left[{\mathrm{Ti}(\mathrm{OR})}_{4}\right]$ is the inorganic precursor concentration, $\eta_{0\_{\mathrm{Ti}(\mathrm{OR})}_{4}}$ is the zero-shear viscosity of the inorganic precursor, $\eta_{0\_\mathrm{PP}}$ is the zero-shear viscosity of PP melt.

According to the Arrhenius law, the kinetic constant of reaction can be written as:

(14) $$k=k_{0}\exp\left(-\frac{E_{a}}{RT}\right)$$

where *k* is the kinetic constant, *k*_0_ is the pre-exponential factor, *E_a_* is the activation energy for reaction, *R* is the gas constant, and *T* is the absolute temperature.

According to Equation (13), the calculated zero-shear viscosity of the mixing fluid with 10wt% precursors and other data are shown in Table 2.

The conversion rate can be expressed as:

(15) $$K_{a}=\;\left(1-\left[\mathrm{Ti}{\left(\mathrm{OR}\right)}_{4}\right]/{\left[\mathrm{Ti}{\left(\mathrm{OR}\right)}_{4}\right]}_{\mathrm{initial}}\right)\times 100\%$$

where K_a_ is conversion rate, [Ti(OR)_4_] is the inorganic precursor concentration at some time, [Ti(OR)_4_]_initial_ is the initial concentration of the inorganic precursor.

### 2.4. Quantitative Mixing 

The area stretch *ζ* is the ratio of the deformed surface *da* at time Δ*t* to initial infinitesimal surface *dA*:

(16) $$\varsigma =\frac{\left|da\right|}{\left|dA\right|}$$

Average mixing efficiency is often used to describe the stretching mixing efficiency during mixing. It is defined as:

(17) $$\langle e_{\varsigma }\rangle =\frac{1}{t}\int_{0}^{t}\frac{\dot{\varsigma }/\varsigma }{{\left(D:D\right)}^{1/2}}dt$$

where $\langle e_{\varsigma }\rangle$ is the time-average mixing efficiency, and **D** is the rate of strain tensor. 

### 2.5. Grid Independence Validation

**Figure 3:**
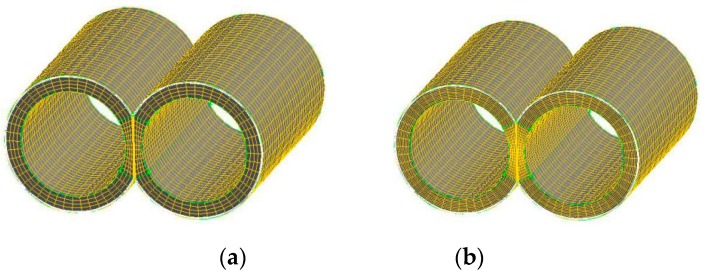
Finite element models with different mesh densities: (**a**) mesh A; (**b**) mesh B.

**Figure 4:**
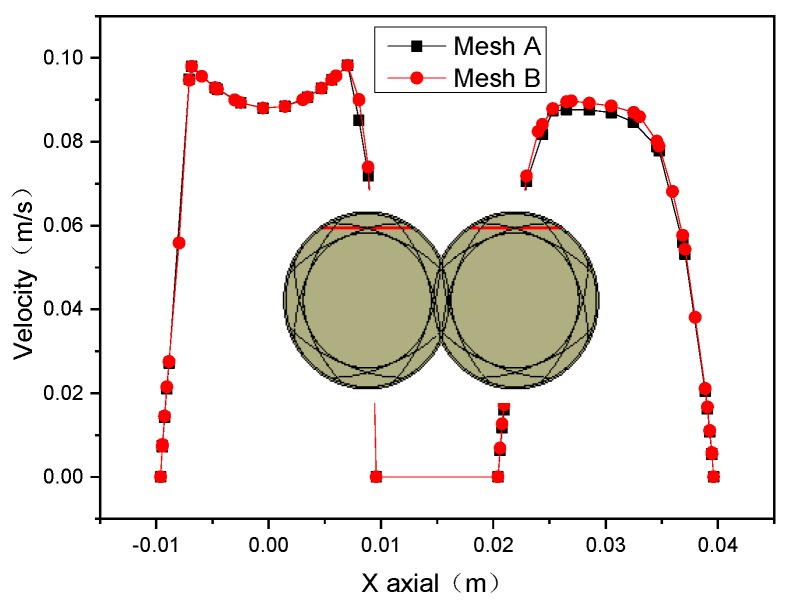
Velocity magnitude profiles and position of line in the TSE.

The effects of cell numbers in the FE model on the numerical results are validated by the grid-independence test. Therefore, two finite element mesh models with different densities are established, as shown in Figure 3, where the model of Mesh A and Mesh B have 34,300 and 57,600 cells, respectively. Figure 4 indicates that the magnitudes of velocity in two kinds of FE models are almost same, which explains that the FE model with 34300 cells meets the requirement of solutions and is selected to study the mixing and reactive mechanisms in the corotating TSE.

## 3. Results and Discussion

### 3.1. Concentration Patterns of Different Species

**Figure 5:**
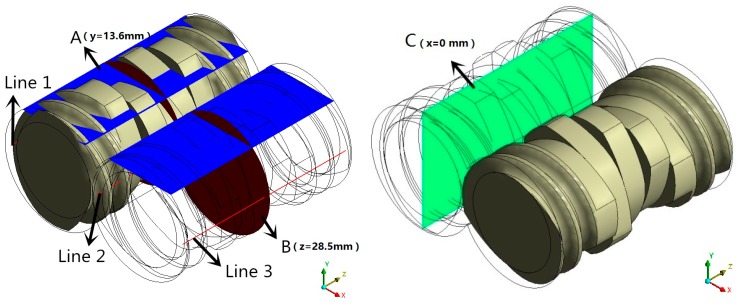
Sketch of sample planes and monitor lines in the TSE.

To learn about the details of mixing and reactive performances in the corotating TSE, some monitor planes and lines are firstly selected. Figure 5 illustrates a sketch of the sample planes and monitor lines of the TSE. The origin of coordinates is at the center of the left screw and three planes are chosen as sample planes, i.e., plane A (*y* = 13.6 mm), plane B (*z* = 28.5 mm) and plane C (*x* = 0 mm), where D = 34 mm is the inner diameter of barrel and L = 57 mm is the axial length of screw. Additionally, Line 1 (*x* = 16 mm, *y* = 0 mm), Line 2 (*x* = 15 mm, *y* = 0 mm) and Line 3 (*x* = 46 mm, *y* = 0 mm) are selected as monitor lines to investigate the local mixing and reactive properties along the axial direction. 

**Figure 6:**
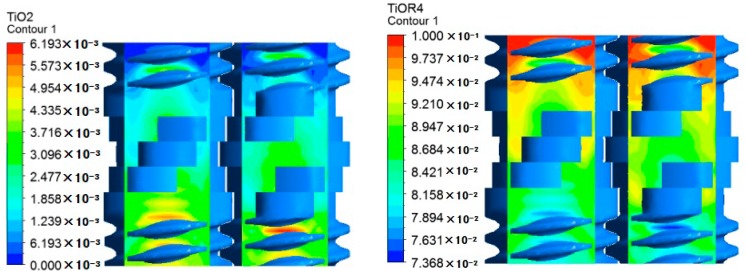
Concentrations of reactant and product at plane A.

Figure 6 shows the concentration distribution of reactant and product at plane A in the corotating TSE, in which the rotational speed is 60 rpm, the stagger angle of the knead blocks is 45°, the initial temperature is 493.15 K and the inlet flow rate is 2 × 10^−6^ m^3^/s. It can be seen from Figure 5, that the concentration of reactant at plane A gradually decreases along the combined screws from inlet to outlet, and the concentration of product tend to go in the opposite trend. In the thread sections, the degree of reaction in the root of thread groove is higher than that in other regions, because the mixing degree of materials in the root of thread groove is relatively high. In the section of kneading blocks, due to the great amplitude of shear rate, the materials blend well, and the distribution of the reaction becomes more uniform.

**Figure 7:**
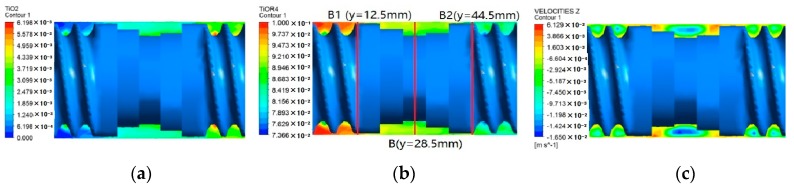
Contours of different fields at cross plane C: (**a**) product; (**b**) reactant; (**c**) axial velocity.

Figure 7 shows the contours of axial velocity, reactant and product at cross plane C of the TSE, in which the left end is the inlet and the right end is the outlet. As can be seen from Figure 7a, the maximum axial velocity occurs at both ends of the kneading block section, resulting in great materials conveyance. The negative axial velocity in the middle area of the kneading block section indicates the presence of back flow, which can enhance the axial mixing capacity and promote the reaction. In Figure 7b,c, the concentration of Ti(OR)_4_ decreased gradually from the inlet to outlet, and the concentration of TiO_2_ has the opposite trend to Ti(OR)_4_. In the kneading block section, due to the presence of back flow, the concentration distributions of reactant and product are more uniform than the thread sections.

**Figure 8:**
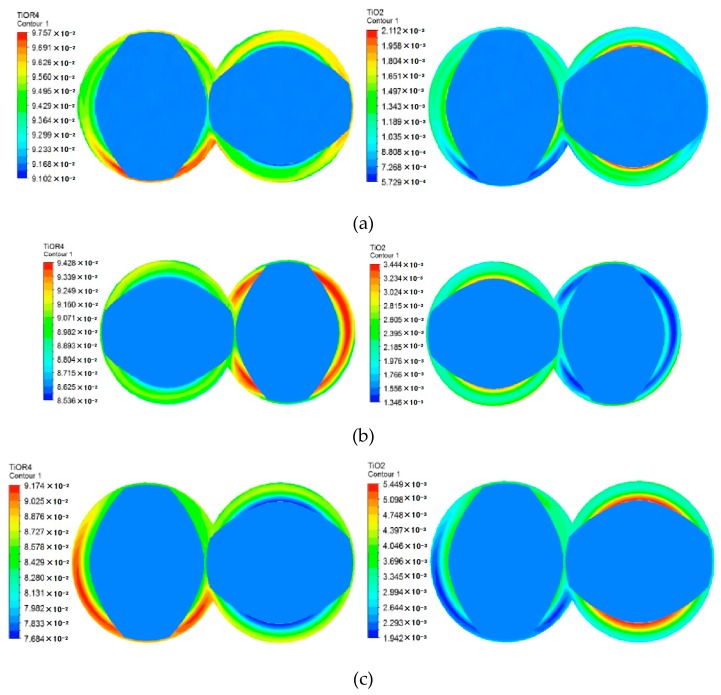
Concentration contours of reactant and product at different axial sections: (**a**) cross plane B1; (**b**) cross plane B; (**c**) cross plane B2.

To accurately study the reaction degree of materials in the axial direction, three planes B1, B, B2 are selected, as shown in Figure 7b. As can be seen from Figure 8, from plane B1 to plane B2, the maximum concentration of reactants decreased from about 0.0976 to 0.0917, and the maximum concentration of TiO_2_ increased from about 0.0021 to 0.0054. Since both planes B1 and B2 are located in the connection between kneading blocks and thread sections, the reaction distribution is roughly the same. Plane B is located in the middle of the kneading block, where the back-flow phenomenon is relatively prominent, so the concentration contours of reactant and product are more uniform than the other two sections.

**Figure 9:**
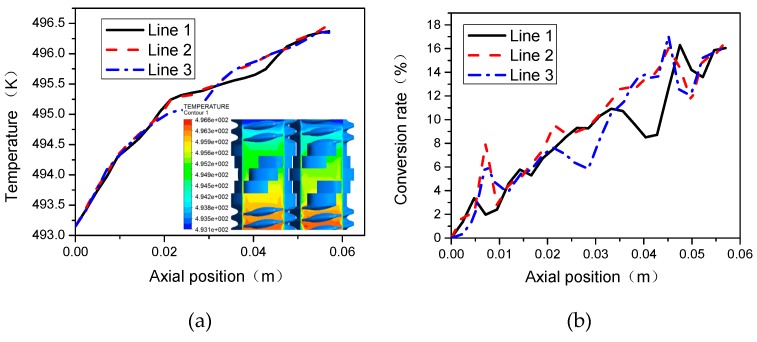
Profiles of temperature and conversion at different monitor lines: (**a**) temperature; (**b**) local conversion rate.

Figure 9 shows the profiles of temperature and conversion at different monitor lines of the TSE. Figure 9a illustrates that the temperature increases gradually from the inlet to the outlet along the axial direction. Since Line 1 and Line 3 are at similar positions, the trends of temperature distributions are roughly the same. Line 2 is located at the intermeshing region of the TSE, where the exchange of material is more frequent and change of temperature is more gradual. It can be seen from Figure 9b that Line 1 and Line 3 have basically the same change trend because their positions are symmetrical in the TSE. Materials in the intermeshing region exchange frequently, leading to good mixing ability and higher temperature amplitude. As a result, the conversion rate at Line 2 is higher than that at Line 1 and Line 3.

### 3.2. Influence of Rotating Speed

**Figure 10:**
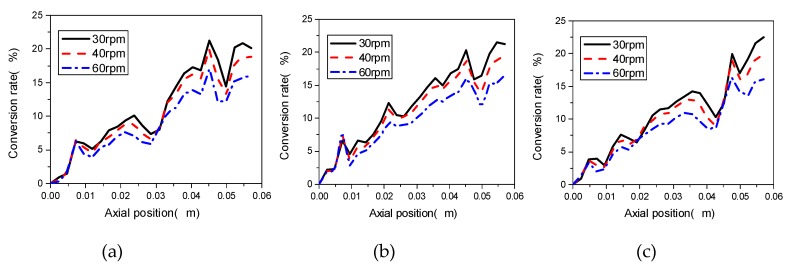
Effect of screw speeds on conversion rate at (**a**) Line 1; (**b**) Line 2; (**c**) Line 3 in the TSE.

The rotational speed of the screw has a direct influence on the conveying ability, mixing effect and conversion rate of the materials in corotating TSEs. Figure 10 illustrates the local conversions at Line 1, Line 2 and Line 3 with different screw speeds, namely 30 rpm, 40 rpm and 60 rpm, in the corotating TSE, in which the stagger angle of the knead blocks is 45°, the initial temperature is 493.15 K and the inlet flow rate is 2 × 10^−6^ m^3^/s. It can be seen that the conversion rates have roughly the same change trend, increasing along the axial distance. Interestingly, the increase of the rotational speed does not promote the reaction. At the same axial position, conversion rate decreases with the increase of speed, this is due to the fact that the increase of screw speed induces the material pass through the TES faster, not leaving enough time for mixing, at the same time reducing the probability of reaction.

To statistically analyze the movement of materials in the composite section of the TSE, the particle trace method (PTM) is adopted based on flow field calculation. 5000 tracer particles without volume, mass and influence on each other were released at the inlet of the corotating TSE and statistical processing is performed using POLYSTAT code embedded into POLYFLOW software. 

**Figure 11:**
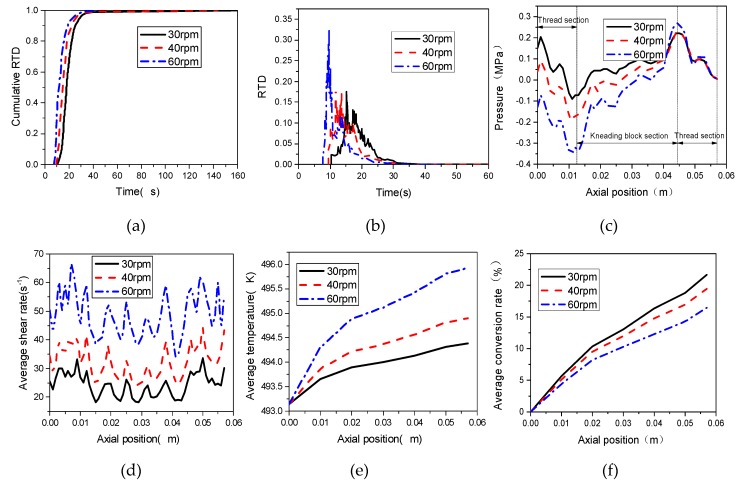
Effect of screw speeds on (**a**) cumulative RTD; (**b**) RTD; (**c**) pressure; (**d**) average shear rate; (**e**) average temperature; (**f**) conversion rate.

Residence time distribution (RTD) is an important parameter for the reactive extrusion process, which directly depends on the rotational speed of screws. Figure 11a illustrates the cumulative RTD distribution of materials in the TSE with different rotational speeds. From Figure 11a, we can see that with the increase of rotational speed, more particles flow out in the same period of time. For example, with 20 s, the cumulative RTD is 0.55 at the rotational speed of 30 rpm, and 0.7, 0.87 at rotational speeds of 40 rpm and 60 rpm, respectively. Figure 11b shows the RTD distribution in the TSEs with different rotating screw speeds. As can be seen from Figure 11b, with the increase of rotational speed, the RTD curves shift from right to left and the transverse width between the inflection points of the RTD curves become narrower gradually, and the average residence time reduced to 21.6 s, 18.16 s and 13.4 s with the increase of rotational speed. This is due to the fact that the increase of screw rotational speed will cause a greater pressure difference between the inlet and outlet of the TSE, as shown in Figure 11c. As is well known, greater pressure difference will decrease the residence of materials, which is not good for the mixing and reaction. 

Figure 11d shows the effect of screw speeds on the distributions of average shear rates along the extrusion direction. It can be seen from Figure 11d that the average shear rates at kneading block section is lower than that in the thread section. With the increase of rotating speed, the shear rate increases gradually at the same axial position, taking the axial position of 0.05 m as example, the average shear rate is about 62 s^−1^ at rotating speed of 60 rpm, 43 s^−1^ at rotating speed of 40 rpm, and 32 s^−1^ at rotating speed of 30 rpm, respectively. In Figure 11e, it can be found that increasing rotating speed improves the average temperature and the variance of average temperature increases dramatically along the axial direction. When the speed is 60 rpm, the maximum temperature magnitude reaches 496 K, about 1 K and 2 K higher than that at speeds of 40 rpm and 30 rpm, respectively. This is due to the fact that the viscous dissipation increases with the increase of rotational speed, which is caused by the increased magnitude of average shear rate, as shown in Figure 11d. The magnitude of average shear rate is important parameters to evaluate the dispersive mixing in TSE. 

According to above analysis, the temperature amplitude of the nanocomposites in the TSE increases with the increase of rotational speed of screws, and reaction rate is positively affected by temperature, so higher rotational speed can promote the progress of the reaction process. However, the average conversion rates decrease with the increase of screw rotational speed, as shown in Figure 11f. This is due to the fact that the improvement of rotation speeds reduces the residence time of materials, which is not conducive to the average conversion rate. 

### 3.3. Effect of Stagger Angle

**Figure 12:**
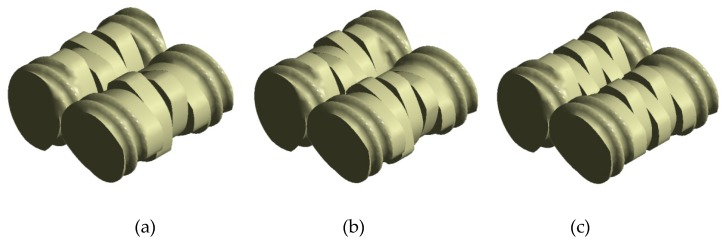
Geometries of screws with different stagger angles of kneading blocks: (**a**) S45; (**b**) S60; (**c**) S90.

The kneading block sections of the combined screws are the dominant ones for mixing efficiency of the whole TSE, greatly influencing the reaction. The stagger angles of kneading blocks have a great impact on the mixing efficiency and conversion rate. In this section, the typical stagger angles of 45°, 60° and 90° of the kneading blocks, namely S45, S60 and S90, as shown in Figure 12, are selected to study the effect of stagger angle on the mixing and reactive characteristics in the TSE. In addition, the rotational speed is 60 rpm, the initial temperature is 493.15 K, and the inlet flow rate is 2 × 10^−6^ m^3^/s.

**Figure 13:**
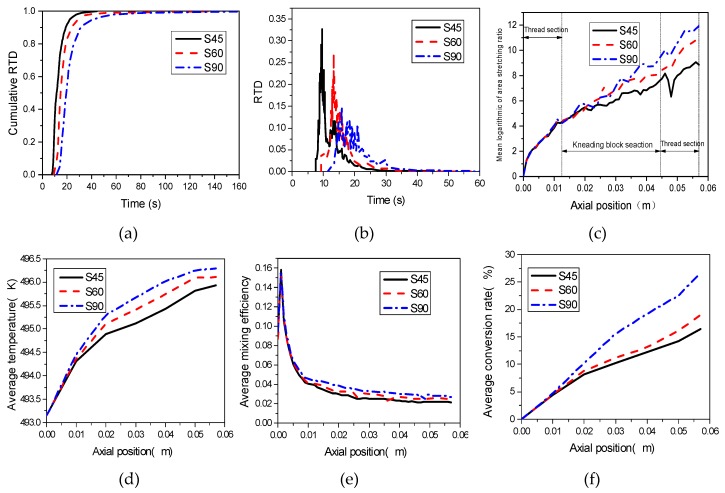
Effect of stagger angle on (**a**) cumulative RTD; (**b**) RTD; (**c**) logarithmic of area stretching; (**d**) mixing efficiency; (**e**) temperature; (**f**) conversion rate.

Figure 13a,b illustrates that increasing the stagger angle of kneading blocks can decelerate the pass through of materials in the TSE. The mean residence times are 13.4 s, 18.97 s and 25.07 s with the increasing of stagger angle, which is conducive to the intensive mixing of the material and the promotion of reaction. 

The mixing efficiency increases with the increase of stretching strength. It can be seen from Figure 13c that the magnitudes of mean logarithmic of area stretching in the three kinds of TESs with stagger angles of 45°, 60° and 90° gradually increase along the axial direction, indicating that they all have good mixing capacity. In the inlet section, the mean logarithmic of area stretching are almost same in three kinds of TSEs. However, in the kneading block section, the magnitude of logarithmic of area stretching in the TSE with stagger angle of 90° is superior to that in the other two TSEs with stagger angles of 45° and 60°, respectively, indicating that screws with a stagger angle of 90° have better tensile capacity and better mixing efficiency, which is advantageous to the reaction.

Under the action of shear rate, viscous dissipation of polymer melt will occur, which will gradually increase the material temperature. As can be seen from Figure 13d, at the same axial position, the temperature increases with the increase of stagger angle. At axial position of 0.04 m, the temperature is 496.1 K when the stagger angle is 90°; as for the kneading blocks with stagger of 60° and 45°, the corresponding temperatures are 495.4 K and 495.8 K, respectively. According to the reaction rate constant of Equation (14), the reaction rate increases with the increase of temperature in the TSE, which is good for the reaction. In addition, Figure 13f provides evidence for this view, at the axial position of 0.04 m, the average conversion rate is about 20% with the stagger angle of 90°, which is much higher than the average conversions of kneading blocks with the stagger angle of 60° and 45°. In addition, the average mixing efficiency increase with the increase of stagger angle, as shown in Figure 13e. Moreover, the variance of the conversion rate is not consistent with the variance of temperature, suggesting that temperature can affect the reaction process, but it is not the only factor which determines the reaction process in the TSE.

### 3.4. Effect of Inlet Flow Rate

The inlet flow rate has an impact on the flow and mixing performances of materials in TSE, and then affects the reaction processes. In this section, we select the TSE with stagger angle of 45°, assuming the rotational speed of screw is 60 rpm, to analyze the effect of inlet flow rate on the preparation process of PP/TiO_2_ nanocomposite.

**Figure 14:**
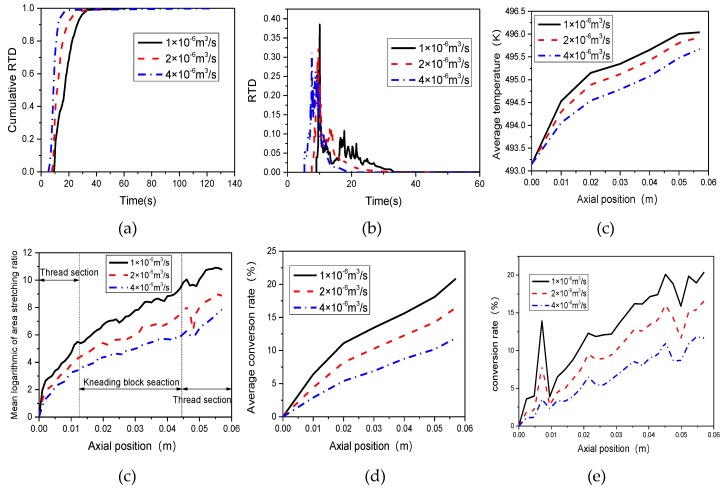
Effect of inlet flow rate on (**a**) cumulative RTD; (**b**) RTD; (**c**) logarithmic of area stretching; (**d**) temperature; (**e**) conversion rate; (**f**) local conversion at Line 2.

Figure 14a,b illustrates the effect of inlet flow rate on the cumulative RTD and RTD, respectively. We can see that increasing the inlet flow rate will decrease the residence time of materials, which is not good for mixing and reaction. The mean residence time is 17.91 s, 13.4 s and 11.55 s with increasing flow rate, as shown in Figure 14b. Figure 14c illustrates the effect of inlet flow rate on the mean logarithmic of area stretching. It indicates that the mean logarithmic of area stretching reduces with the increase of inlet flow rate, which is averse to mixing of materials. Figure 14d depicts that the average temperature increases along the axial direction, and increasing the inlet flow rate will reduce the average temperature. According to Equation (14), the reaction rate constant increases with the increase of temperature magnitude, indicating that higher inlet flow rate is not conducive to the proceeding of reaction. As can be seen from Figure 14e, the average conversion rate decreases with the increase of inlet flow rate, which further prove the conclusion above. Figure 14f shows the effect of inlet flow rate on local conversion at the monitor Line 2 of the TSE. 

### 3.5. Effect of Initial Temperature

Temperature has a significant influence on material diffusion, convection and reaction rate. We select the TSE with a stagger angle of 45°, and a speed of screw of 60 rpm, to analyze the effect of initial temperature of barrel on the preparation process of PP/TiO_2_ nanocomposite. Line 2 is selected as the local analysis object, and the initial temperature of barrel in the corotating TSE is set as 453.15 K, 493.15 K and 523.15 K, respectively.

**Figure 15:**
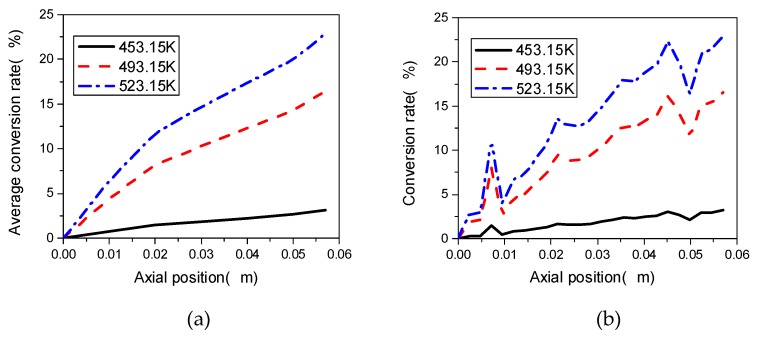
Effect of initial temperature of barrel on conversion rate at (**a**) Line 2; (**b**) the whole TSE.

Figure 15a shows the change curve of local conversion rate at Line 2 of the TSE at different initial temperatures of barrel. As can be seen from the figure, the change trends of the conversion rate at the monitoring Line 2 are roughly same. Along the axial direction of the screws, the local conversion rate of Line 2 gradually increases from the inlet to outlet of the TES, which fluctuates at both ends of the combined screws, and steadily rises in the middle meshing block area. At the same axial position, the higher the initial temperature is, the higher the conversion rate obtains. When the initial temperature in the TSE is 453.15 K, the reaction is relatively slow, and the highest conversion rate is only about 3%. However, under the other two initial temperatures, the conversion rate at Line 2 reaches 17% and 23% respectively, with a large difference.

Figure 15b shows the distribution of average conversion rate change in the TSE at different initial temperatures of barrel. It can be seen from this figure that the reaction is slow at low initial temperature magnitude. For example, when the initial temperature magnitude is 523.15 K, the average conversion rate reaches 23%, which is about 20% higher than that when the initial temperature is 453.15 K. This phenomenon is mainly due to the fact that the reaction rate constant is greatly affected by temperature distributions in the TSE.

## 4. Conclusions

In this work, the sol-gel reaction process of preparing PP/TiO_2_ nanocomposites in a corotating TSE with two combined screws of conveying and mixing screw elements is first investigated via 3D numerical modeling based on FEM. The reactive patterns of nanocomposites in the corotating TSE are firstly investigated. Moreover, to control reactions in extrusion and optimize the geometric parameters of TSE, the effects of screw rotational speed, stagger angle, inlet flow rate and initial barrel temperature on the mixing and reaction process are investigated, and the relationship between the mixing characteristics and the reactive process in a corotating TSE is discussed. The following conclusions are drawn.

In the reaction of PP/TiO_2_ nanocomposites prepared by a corotating TSE, the conversion increases gradually from the inlet to the outlet along the axial position. Although increasing the rotating speed of the screw can increase the average temperature of materials, as the axial velocity of materials increases, the residence time of the materials in the TSE gradually becomes shorter, which is not conducive to the full mixing and reaction process of materials. Increasing the stagger angle of the kneading blocks gradually increase the temperature of the material which is good to improve the conversion rate. 

At the same time, increasing stagger angle will improve the stretching efficiency of the material in TES, and prolong the residence time of the material in the extruder, which is conducive to the intensive mixing and reaction. Increasing the flow rate at the entrance of the screw extruder in the combination sections will shorten the residence time of the material in the extruder, which is not conducive to mixing. 

Moreover, the temperature change is inversely proportional to the inlet flow rate, and the conversion rate decreases with the increase of the inlet flow rate. Increasing the initial temperature in the TSE will reduce the viscosity of the material while increasing the reaction rate constant, speed up the reaction process, and gradually increase the conversion of PP/TiO_2_ nanocomposites. Generally, besides the parameters mentioned above in this paper, concentration of the precursor and the length of the screw will greatly influence the mixing and reaction processes in the TSEs. Consequently, study of these two parameters will provide reference for the control and optimization of reactive extrusion processes in TSEs. 

## Figures and Tables

**Table 1:** Parameters of geometric model of the extruder.

Parameter	Value
Inner diameter of barrel	33 mm
Screw tip diameter	32 mm
Screw root diameter	26 mm
Centerline distance of screws	30 mm
Screw clearance	0.5 mm
Clearance of screw and barrel	0.5 mm
Leads of screws	2
Length of kneading blocks	32 mm
Length of thread sections	12.5 mm
Total length of combined screws	57 mm

**Table 2:** The material properties at different temperature.

[Ti(OR)_4_] wt%	$k(s^{-1})$ 453.15 K	$k(s^{-1})$ 493.15 K	$k(s^{-1})$ 523.15 K	Ea (KJ·mol^−1^)	$\eta_{0}\;(\mathrm{Pa}\cdot S)$
10 wt%	0.0017	0.0102	0.0152	65	3500
